# Supplementary material for: Persistent Immune Dysregulation during Long COVID is Manifested in Antibodies Targeting Envelope and Nucleocapsid Proteins
Source: Res Sq. 2026 Jan 8:rs.3.rs-8302624. Preprint. [Version 1] doi: 10.21203/rs.3.rs-8302624/v1 (PMC12803341; doi:10.21203/rs.3.rs-8302624/v1)
Supplement: 1 [file NIHPPRS8302624V1-supplement-1.pdf]

Supplementary Table 1. Extended summary of general health assessment for participants enrolled in the study.

Characteristic	Level	CONV	LC
General Health Level	Excellent	3 (30%)	1 (5%)
	Very good	5 (50%)	6 (30%)
	Good	2 (20%)	10 (50%)
	Fair	0 (0%)	1 (5%)
	Poor	0 (0%)	2 (10%)
Quality of Life Level (	Excellent	4 (40%)	3 (15%)
	Very good	4 (40%)	8 (40%)
	Good	2 (20%)	8 (40%)
	Fair	0 (0%)	1 (5%)
	Poor	0 (0%)	0 (0%)
Physical Health Level	Excellent	3 (30%)	0 (0%)
	Very good	5 (50%)	4 (20%)
	Good	2 (20%)	13 (65%)
	Fair	0 (0%)	1 (5%)
	Poor	0 (0%)	2 (10%)
Fatigue Level	None	3 (30%)	0 (0%)
	Mild	3 (30%)	3 (15%)
	Moderate	4 (40%)	13 (65%)
	Severe	0 (0%)	4 (20%)
	Very severe	0 (0%)	0 (0%)
Sleep Refreshing Level	Not at all	0 (0%)	1 (11%)
	A little bit	0 (0%)	5 (56%)
	Somewhat	1 (100%)	2 (22%)
	Quite a bit	0 (0%)	1 (11%)
	Very much	0 (0%)	0 (0%)
	Missing	9	11
Emergency Visit	Yes	0 (0%)	2 (10%)
Hospital Visit	Yes	0 (0%)	1 (5%)
